# Knowledge about the Care of People with Alzheimer’s Disease of the Nursing Staff of Nursing Homes in Spain

**DOI:** 10.3390/ijerph16244907

**Published:** 2019-12-05

**Authors:** Laura Parra-Anguita, Francisco P. García-Fernández, Rafael del-Pino-Casado, Pedro L. Pancorbo-Hidalgo

**Affiliations:** Department of Nursing, Faculty of Health Sciences, University of Jaén, 23071 Jaén, Spain; fpgarcia@ujaen.es (F.P.G.-F.); rdelpino@ujaen.es (R.d.-P.-C.); pancorbo@ujaen.es (P.L.P.-H.)

**Keywords:** knowledge, nursing home, Alzheimer’s disease, dementia, nursing staff

## Abstract

People with Alzheimer’s disease often live in nursing homes. Updated knowledge among the nursing staff has led to better quality of care. The aim of this study was to measure the knowledge about the care of people with Alzheimer’s disease of the nursing staff of nursing homes in Spain. A cross-sectional study was conducted in 24 nursing homes in the province of Jaén (Spain) with a sample of 361 members of staff, i.e., registered nurses (RNs), assistant nurses (ANs), and eldercare workers (EWs). The University of Jaén UJA-Alzheimer’s Care Scale was used to measure the knowledge. The knowledge was higher among the RNs (83.3% of the maximum) than among the ANs and EWs (71.6%). Work experience and updated training were associated with the knowledge score in RNs, but only the updated training in ANs and EWs. Nursing homes with less experienced nursing staff and with a small proportion of staff receiving training on dementia have a low knowledge score. The nursing staff of nursing homes in Jaén have medium to high knowledge about Alzheimer’s care. There is a wide range of variation in the knowledge score among the nursing homes. Up-to-date staff training in dementia care is the factor with the strongest association with knowledge.

## 1. Introduction

According to the World Health Organization, dementia is one of the main health problems affecting older people [1]. The most common form of dementia worldwide is Alzheimer’s disease (AD) (50–70% of cases) [2], followed by vascular dementia (12.5–25%) [3,4]. In Spain, approximately 600,000 people suffer from any type of dementia, of whom 400,000 suffer from Alzheimer’s disease [5]. The prevalence of dementia ranges from 5% to 17.2% [5,6,7].

In the advanced stages of Alzheimer’s disease (AD), the care required is more complex, so high-quality care can often only be provided in institutions such as nursing homes. As a result, many elderly people with AD are admitted [8]. In Spain, two-thirds of the elderly people admitted to nursing homes suffer from dementia [9].

Optimal dementia care in nursing homes adopts a person-centered perspective that focuses on maximizing residents’ quality of life [10]. Nursing homes play an important role in providing care for dependent elderly people, so ensuring high quality of care for residents in these settings is essential. Poor-quality care has been associated with inadequate nursing staffing and a poor skill mix [11].

In nursing homes, quality has two dimensions: quality of care and quality of life. Quality of care refers to more technical aspects of care (such as the use of restraints or the prevalence of pressure ulcers) while quality of life refers to issues such as residents’ autonomy or decision-making capacity [11]. The most commonly used indicators of quality of care are pressure ulcers, use of restraints, functional status, mortality, hospitalization, nutritional status, incontinence, etc. [11]. Factors such as depression, conduct disorders, and functional limitation impair quality of life [10]. The quality of care is not only determined by the staff ratio but also by other factors such as management practices, the education level of the nurses, proper staffing, care planning, and recruitment and retention issues [10,11].

Until now, research in this area has tended to focus on measuring quality in relation to the number of nurses. Other determinants, such as turnover, workers’ stability, staff training and care management, may affect the staff’s performance and their relationship to the quality of care provided to residents [11].

Nurses and health-care workers who care for people with dementia must receive specific training to provide high-quality care [12]. Several studies have determined the knowledge that health-care professionals have about AD and other dementias, finding that registered nurses (RNs) have greater knowledge than assistant nurses (ANs) [13,14]. In addition, among the staff who provide care to people with dementia in nursing homes, there is great variability in terms of academic education and training [15]. Some studies found that usually the staff providing 90% of basic care to people with dementia have low qualifications (ANs or eldercare workers (EWs)) [16].

Knowledge about dementia is determined by education and training [17], but also by direct practical experience when caring for people with dementia [18]. Nursing home managers should promote continuous education and in-service training activities aimed at engaging the staff [19,20]. Training positively influences staff confidence, improving their performance and the quality of care provided [21].

Several studies have addressed the issue of nurses and other health-care workers caring for people with dementia. In Portugal, a qualitative study found that nurses in hospitals have limited knowledge about the characterization of AD, risk factors, diagnostic means, complications, and drugs for the treatment and care of patients and carers [22]. Another study in Australia concluded that health workers had moderate knowledge, but there were significant differences between workers with and without previous experience in caring for people with dementia [12]. Robinson et al. compared the knowledge about dementia care among nurses, nursing assistants, and family members. Surprisingly, some family members have greater knowledge of dementia than RNs [23].

Therefore, the literature shows that it is important to know the level of knowledge about AD and dementia of the nurses and care workers who care for people with dementia. The aims of this study were: (a) to determine the knowledge of staff in nursing homes in Spain about evidence-based practices in caring for people with AD; and (b) to analyze which factors are associated with this knowledge.

## 2. Materials and Methods

### 2.1. Design

A cross-sectional study was conducted in 24 nursing homes (5 public and 19 private) located in the province of Jaén (southern Spain). Twenty-six nursing homes were contacted, two of which refused to participate; this is 33% of the total number of 72 nursing homes in the province. The staff of nursing homes is mainly composed of registered nurses, assistant nurses, and eldercare workers, as well as some physiotherapists, social workers, and occupational therapists. Only two of the nursing homes had a doctor on the staff; the others had doctors providing services a few hours some days of the week.

The STROBE guidelines were used for reporting this study [24].

### 2.2. Study Population and Sample

The convenience sample consisted of 361 nursing professionals, i.e., registered nurses (RNs, four-year university degree), assistant nurses (ANs, two-year diploma) and eldercare workers (EWs, one-year technical education) working in 24 nursing homes.

The inclusion criteria were: (1) being registered nurses, assistant nurses, or eldercare workers; and (2) participating voluntarily.

### 2.3. Variables and Instruments

The data were collected by using a self-administered form with two sections:

- Socio-demographic variables: gender, age, and experience
- Education and training: attendance at courses and conferences

The knowledge about caring for people with AD and other dementias was measured using the UJA-Alzheimer’s Care Scale (Spanish version) (UJA stands for University of Jaén) [25]. This is a 23-item questionnaire with three response options: “Yes”, “No”, and “I don’t know”. Correct answers are scored with 1 point; errors and “don’t know” with 0 points. Total score: 0–23 (more points means more knowledge). This questionnaire has good psychometric characteristics among Spanish nursing home staff: internal consistency (α = 0.70), test-retest reliability (intraclass correlation coefficient = 0.83), and convergent validity with the Spanish version of the Dementia Knowledge Assessment Tool 2 (DKAT2-Sp) [26] (ICC = 0.62). It is easy to administer and can usually be completed in 5–10 min.

### 2.4. Data Collection

Data collection was conducted from November 2016 to January 2017. The research protocol was approved by the Jaén Research Ethics Committee (project code 25102012).

First, we contacted the directors of the nursing homes to request collaboration and the authorization to administer the questionnaires to the staff of these centers. Then, each center received a number of printed questionnaires in accordance with the number of people on their staff. To guarantee anonymity, no personal data were included in the questionnaire.

### 2.5. Data Analysis

The characteristics of participants were analyzed using descriptive statistics, frequencies and percentages for categorical variables, and mean and standard deviation for continuous variables. To obtain knowledge scores, the mean, standard deviation, 95% confidence interval (CI), and the percentages of correct, incorrect, and “don’t know” answers were calculated. The final score of the scale corresponds to the number of correct answers. In addition, the count and percentage of errors (incorrect answers) and “don’t know” (explicitly marked) were calculated. The option “don’t know” allows identifying the gaps in knowledge.

The association between demographic or educational variables and the knowledge score was tested using the Student’s t-test or one-way ANOVA. The effect size was estimated using the Cohen’s d statistic. A multivariate analysis by stepwise linear regression was carried out with the variables with statistical significance in the bivariate analysis. Analyses were performed with SPSS 21 (IBM^®^ International Business Machines Corporation, Armonk, NY, USA). A value of *p* < 0.05 was used as the level of statistical significance.

## 3. Results

### 3.1. Study Sample Characteristics

In total, 361 questionnaires valid for analysis (51.5% response rate) were obtained from the 24 nursing homes surveyed. Table 1 shows the main characteristics of the participants.

### 3.2. Knowledge about Alzheimer’s Disease

The knowledge about the care of people with AD as measured with the UJA Alzheimer’s Care Scale is shown in Table 2. Knowledge scores are significantly higher for RNs than for ANs and EWs together (*t* = 6.86; *p* < 0.0001) with a large effect size (Cohen’s d = 0.94; CI 95% 0.66–1.21) and lower for error and “don’t know” scores, respectively (*t* = −5.17; *p* < 0.0001; Cohen’s d = 0.71; CI 95% 0.43–0.98) (*t* = −3.95; *p* < 0.0001; Cohen’s d = 0.54; CI 95% 0.27–0.81). The RNs obtained 83.30% of correct answers, 11.5% errors, and 4.3% for “don’t know”. In the group of ANs and EWs, the percentages obtained were 71.6%, 18.2%, and 9.8% for correct, errors, and “don’t know”, respectively. Overall, these values mean that RNs obtained a 12% higher score than ANs and EWs.

Table 3 shows the percentages of correct, incorrect, and “don’t know” answers for the items of the UJA Alzheimer’s Care Scale, ordered from more to less. In the RNs group there are nine very well-known items (>90% of correct answers), 11 well-known (between 70% and 90%), and three items with moderate knowledge (between 50% and 70%). In the ANs and EWs group, there are six very well-known items, seven well-known items, five moderately well-known items, and four little-known items (less than 50%).

### 3.3. Factors Associated with Knowledge about Alzheimer’s Care

The association between the score on the UJA Alzheimer’s Care Scale and several demographic and educational factors was tested. The age of the professionals did not correlate with the knowledge score, either in RNs (*r* = 0.195; *p* = 0.113) or in ANs and EWs (*r* = 0.091; *p* = 0.142). Table 4 shows a comparison of the mean knowledge scores according to the variables tested, in both groups.

The variables that showed a statistically significant association with the knowledge score in the bivariate analysis were adjusted using a linear regression model (stepwise model). Linearity, residual independence, homoscedasticity, and non-collinearity were tested. Model fit was tested by R^2^. In the group of RNs, two variables remained with statistically significant association after adjustment: work experience (beta = 0.393; *p* = 0.001) and attendance at courses on dementia in last three years (beta = 0.256; *p* = 0.034). In the group of ANs and EWs, only attendance at courses on dementia in the last three years remains significant (beta = 0.182; *p* = 0.011).

### 3.4. Knowledge about AD Care at Nursing Home Level

**Figure 1:**
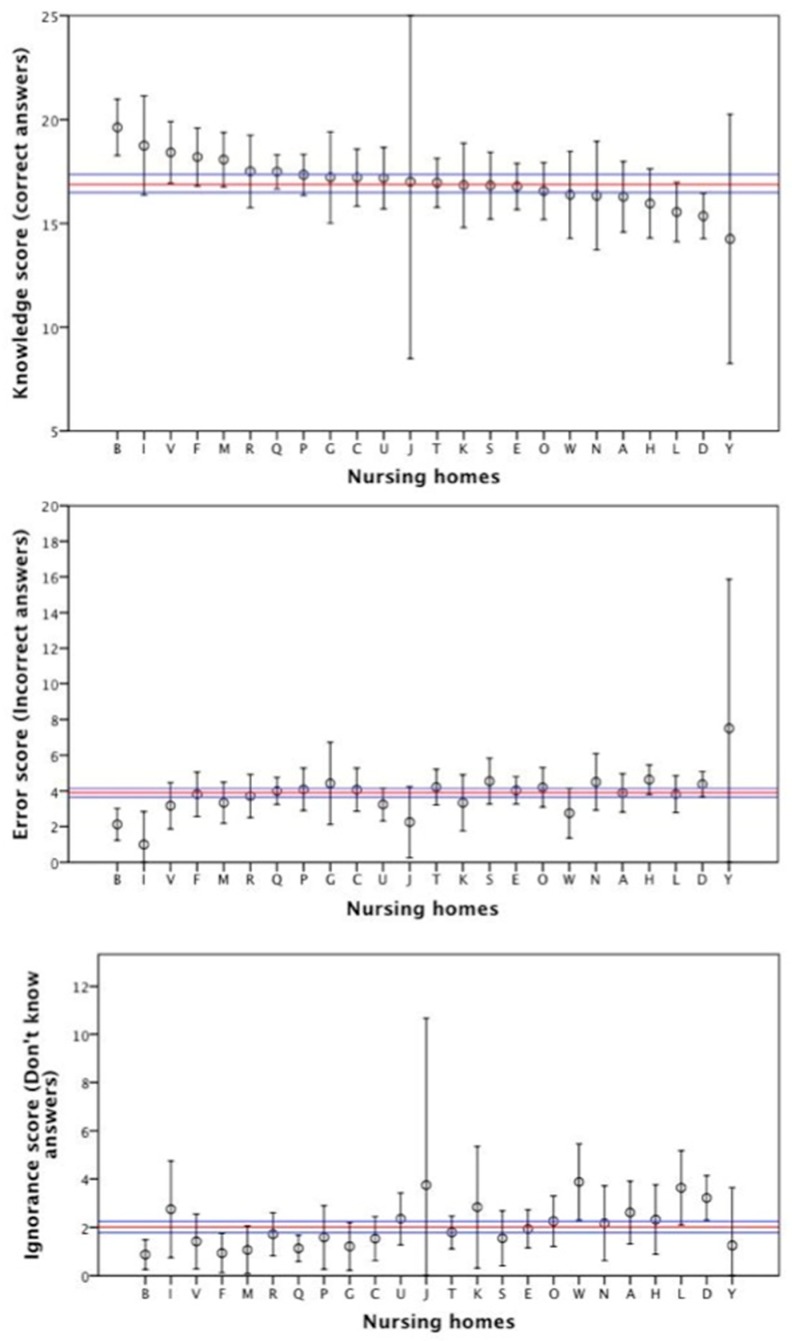
Knowledge score of the nursing homes.

At the level of each nursing home, we found great variability in the staff knowledge about AD care, according to the mean score on the UJA Alzheimer’s Care Scale. Figure 1 shows the mean scores of the 24 nursing homes surveyed ranked in knowledge score decreasing order. Eight nursing homes scored above the upper limit of the CI95% of the overall mean (staff with high knowledge) and six scored under the lower limit (staff with poor knowledge). Most of the nursing homes with the highest knowledge scores have the lowest errors and ignorance scores. Nevertheless, there is a strong negative correlation between errors and ignorance scores, after adjustment for knowledge score (*r* = −0.96; *p* < 0.0001), thus, in nursing homes where staff do not recognize that they do not know the answers for certain items, they had more errors.

To explain the variability found in the knowledge scores among the nursing homes, some of their characteristics were analyzed. Nursing homes were classified according to: the number of residents; the experience of the staff members (percentage of RNs, ANs and EWs with more than 5 years or more than 15 years of experience); updating of training (percentage of staff who have attended dementia courses in the last three years); and the ratio of residents per staff member (median = 2.90, minimum = 1.14, and maximum = 7.50). Table 5 shows the characteristics of the 24 nursing homes and their association with the mean knowledge score obtained in each nursing home. Neither the size of the nursing home (number of residents) nor the ratio of residents per staff member was associated with the knowledge score. The experience of the staff and the updating of training were associated. Nursing homes with a very inexperienced staff (less than 33% having more than five years of experience) scored lower than the rest (moderate effect size). In addition, nursing homes with a high percentage of staff having recently attended dementia courses scored higher (moderate effect size).

## 4. Discussion

The results of our study show that the staff of the nursing homes in the province of Jaén has a medium to high level of knowledge about the care of people with Alzheimer’s disease and other dementias. The knowledge is high among RNs (83.3% of the maximum score on the UJA Alzheimer’s Care Scale) and slightly lower among ANs and EWs (71.3%). This difference in the knowledge score can be explained by the difference in education, although the score obtained in the group of ANs and EWs is higher than expected. These results are quite similar to those reported by other researchers. Smyth et al. [12], in the health service of the Queensland area in Australia, found that the knowledge score regarding AD was higher in professional categories with more years of education (physicians 86.6%; nurses 79.6%) and lower among ANs and support staff. Robinson et al. [23] measured knowledge using the Dementia Knowledge Assessment Tool 2 (DKAT2) [27] in a sample of nursing staff and family members in nursing homes, reporting that nurses scored higher than ANs. Similar findings were reported by Annear et al. [28] in a study aimed at measuring the knowledge of nurses and assistant staff before and after a specific training program. They concluded that nurses have a higher level of knowledge than assistant staff. A study in a different cultural context, namely China, reported a moderate knowledge score in a group of non-professional carers in nursing homes (17.4 points out of 23) [29], which is similar to the score for ANs and EWs in our study.

In our results, both groups (RNs and ANs and EWs) showed a high degree of coincidence in the items best known, such as “recording of mechanical containment”, “information to the carer”, “inclusion of patients’ representatives in decision-making”, and “recording in the clinical history of the behavioral and psychological symptoms of dementias”. In addition, there are coincidences in the worst-known items: “use of nasogastric tube and percutaneous gastrostomy” and “treatment of psychological and behavioral disorders”. We emphasize that the item on the measurement of carer overload by means of the Zarit scale has the highest self-declared ignorance (percentage of response “I don’t know” for RNs: 14.5% and ANs: 55.8%). Comparatively, in the study by Robinson et al. [23], the best-known aspects were those related to the physiopathology, behavioral and psychological symptoms of dementias, and decision-making; and the least-known ones were those related to palliative care and the terminal phase of the disease. Our findings agree with other authors [30] on the poor knowledge of staff about the topic of the management of behavior disorder. Other topics mentioned in terms of low knowledge were: effective communication strategies with patients; early recognition of dementia; and non-pharmacological treatment or epidemiology [30].

It is important to emphasize that the identification of knowledge gaps among nursing staff through well-designed questionnaires makes it possible to organize staff training according to these gaps. Specialized training increases knowledge and improves the quality of care provided [28]. Adequate training in AD care allows situations of bad care to be avoided in different areas such as pain treatment in people with dementia [31], as well as unnecessary or futile tests and interventions in the last three months of life [23]. Adequate recording of the use of mechanical restraints when needed is a well-known item in our findings. This is an important issue, with a high impact in clinical practice, as shown by some studies [32].

Having updated training in dementia, measured by attendance at courses in the last three years, is the single most important factor associated with a high level of knowledge among nursing staff (RNs and ANs). This is in line with results found in studies on specialized training [12,28]. The second factor associated with knowledge is the number of years of experience in nursing, but only within the RNs group. Both inadequate training and a lack of experience have been reported to be negatively associated with the quality of care provided by nurses [30]. In our study, we did not find that the age of the staff was associated with knowledge, in contrast to the findings of other authors [12].

In regard to the scores of each nursing home, we found a wide range of variation in the knowledge score. We found a small group of nursing homes with nursing staff scoring higher than average and another group scoring lower. Although our study was not specifically designed to investigate this difference, we tested some factors that could explain it, at least partially. Again, training and experience are associated with greater knowledge. Our findings show that those nursing homes with a higher percentage of nursing staff that have attended training courses recently (last three years) had a high level of knowledge. The same occurs in nursing homes where more than 33% of their staff has more than five years of experience. In contrast, we did not find any association between knowledge score and the size of the nursing home or with the ratio of residents to staff. Overall, our results support the theory that the quality of care in nursing homes is influenced by a variety of factors and not just by staffing [11] or size [30]. Factors such as staff training and professional experience influence the level of knowledge, which in turn influences the quality of care. Training programs lead to nurses having more positive attitudes and increase their satisfaction with the care of people with dementia [30].

Research findings suggest that nursing home managers should evaluate the knowledge of the nursing staff about dementia care as a first step in planning and developing training activities. Several studies have shown an increase in the level of knowledge after developing training programs [33,34,35]. Some authors, e.g. Gaugler et al. [10], advocate that institutions should develop incentives, scholarships, and other initiatives to expand the training of staff. Other authors, e.g. Matsuda et al. [35], propose training programs with good results. The program consists of two 90-min training sessions (on different days) developing topics such as types of dementia, symptoms, behavioral problems, early detection and treatment, prevention, and the attitude of professionals towards people with dementia. We emphasize the great importance of continuous training in nursing clinical practice, because a higher level of knowledge is related to a higher quality of care [11] and less difficulty for professionals in interacting with people with dementia [35]. Nursing staff of nursing homes must be trained to respond to the needs of their patients, adapting and updating themselves for person-centered care. The tool used in this study could be used to develop research in other settings, such as hospitals (where people with dementia are treated quite frequently) or community care to identify the training needs of nurses and healthcare providers.

This research has some limitations that should be pointed out. Data were obtained through self-administered questionnaires, so some information could be shared when filling them in. Sampling was not random and some nursing homes had a low response rate, thus it is possible that the most motivated staff predominantly responded, which may have led to an overestimation bias in the scores. We tried to overcome these limitations with the design of a multi-center study, including 24 nursing homes, thereby increasing the representativeness of the sample. In addition, the sample size of the group of RN is small for multivariate analysis and reduces its reliability. Finally, it is important to bear in mind that the questionnaire used has a “Don’t know” option for each item that allows independent analysis of errors and ignorance, as recommended by other authors [23].

## 5. Conclusions

The nursing staff working in nursing homes in the province of Jaén has a medium to high knowledge about Alzheimer’s disease care. The knowledge score is higher among RNs than among ANs and EWs. Nevertheless, there is a wide range of variation in the mean score at the level of each nursing home, with a small group of nursing homes having higher than average scores. Future research should be aimed at identifying the factors that explain this variability. The knowledge score of the RNs is associated with the years of working experience and with receiving updated training in dementia. For ANs and EWs, only receiving updated training is associated with the knowledge score. Nursing staff of nursing homes must be trained to respond to the needs of their patients, adapting and updating themselves in the new person-centered approaches to care.

## Figures and Tables

**Table 1:** Socio-demographic characteristics of the nursing home staff.

Variables	Frequency (%)	
	RNs (*n* = 69)	ANs and EWs * (*n* = 292)
Age; mean (SD)	33.21 (8.78)	38.87 (10.31)
Gender		
Female	65 (94.2%)	275 (94.2%)
Male	4 (5.8%)	15 (5.1%)
Work experience (years)		
<5	25 (36.2%)	111 (38%)
5–15	26 (37.7%)	149 (51%)
>15	18 (26.1%)	31 (10.6%)
Attendance at courses on dementia (any time)		
Yes	61 (88.4%)	227 (77.7%)
No	8 (11.6%)	64 (21.9%)
Attendance at courses on dementia (last 3 years)		
Yes	31 (44.9%)	106 (36.3%)
No	21 (30.4%)	87 (29.8%)
Attendance at conferences or meetings (any time)		
Yes	27 (39.1%)	79 (27.1%)
No	42 (60.9%)	203 (69.5%)

* RNs, registered nurses; ANs, assistant nurses; EWs, eldercare workers. The percentage missing from the variables up to 100% corresponds to the no answers.

**Table 2:** Knowledge of nursing home staff about caring for people with AD.

Scores	Mean (SD); CI 95%	
	RNs (*n* = 69)	ANs and EWs * (*n* = 292)
Knowledge score	19.16 (2.48); 18.56–19.76	16.46 (2.91); 16.08–16.76
Error score	2.65 (1.95); 2.18–3.12	4.19 (2.32); 3.92–4.46
Don’t know score	0.99 (1.54); 0.61–1.36	2.25 (2.28); 1.98–2.51

* RNs, registered nurses; ANs, assistant nurses; EWs, eldercare workers.

**Table 3:** Percentage of correct answers, errors and “don’t knows” for the items of the UJA Alzheimer’s Care Scale.

UJA Alzheimer’s Care Scale	RNs			ANs and EWs		
	Correct	Errors	“Don’t Know”	Correct	Errors	“Don’t Know”
9 ^a^. The application of mechanical restraints, type and date of application, reason, care provided and informed consent should be recorded in the patient medical record.	100%			93.5%	3.1%	3.4%
14. Inform the carer about the disease and its possible complications, and the social resources and support systems available.	100%			92.1%	5.8%	1.4%
10. Palliative care must include psychosocial, spiritual, cultural and family support aspects.	98.6%	1.4%		89%	6.5%	4.5%
16. Identify who is the patient’s representative to include him or her in decision-making and care planning.	98.6%			92.1%	5.8%	2.1%
21. Record in the patient medical record data on the form of onset, progression, psychological and behavioral symptoms.	97.1%	2.9%		91.4%	5.5%	3.1%
5. Non-pharmacological and pharmacological measures should be used together to manage the different behavioral and psychological symptoms of dementia.	95.7%	2.9%	1.4%	78.1%	7.9%	13%
22. Care plans should address activities of daily living to maximize independent activity, maintain function, and adapt and develop skills.	95.7%	2.9%	1.4%	93.5%	1.7%	4.1%
6. Reporting the existence or suspicion of abuse is not a matter for nurses or elderly care workers, but for other professionals.	94.2%	2.9%	1.4%	68.8%	27.1%	3.8%
19. Provide comprehensive care to the carer, including counselling and emotional support.	94.2%	1.4%	2.9%	92.5%	3.4%	3.8%
17. Carers should be informed and trained to prevent the onset of behavioral and psychological symptoms of dementia.	89.9%	5.8%	1.4%	88.7%	7.9%	3.1%
7. The management of extreme agitation, violence and aggressiveness must take place in a safe, low-stimulation environment, separate from other users of the service.	87%	5.8%	5.8%	78.4%	15.4%	5.1%
11. Conduct long-term physical activity programs to maintain the functional capacity of institutionalized dementia patients.	87%	7.2%	2.9%	87.7%	6.2%	5.5%
4. Provide a normal diet, while assessing the causes of dysphagia.	81.2%	10.1%	8.7%	68.8%	20.5%	10.3%
2. The Zarit scale is used to quantify the carer’s burden.	78.3%	5.8%	14.5%	31.5%	9.6%	55.8%
12. Use the oral route for fluid supply at the end of life, whenever possible.	76.8%	18.8%	4.3%	83.2%	11.3%	4.8%
15. Behavior modification, programmed hygiene and induced micturition increase urinary incontinence in patients with dementia.	76.8%	15.9%	7.2%	44.5%	39.4%	15.1%
1. If needed, mechanical restraints can be used as a substitute for surveillance or for the convenience of professionals.	79.3%	21.7%	4.3%	63.7%	30.1%	5.5%
20. Intervention programs in activities of daily living do not reduce the carer’s burden in the medium term.	73.9%	17.4%	7.2%	52.1%	31.8%	15.8%
3. When families cannot guarantee care for people with dementia, admission to a facility may avoid social isolation and prevent abuse.	72.5%	20.3%	4.3%	70.9%	25.7%	2.7%
18. Advise the person with dementia to prepare a living will document in the early stages of the disease.	71%	17.4%	11.6%	54.8%	23.3%	21.2%
13. Informing family and carers of the near death status does not improve care in the last few days.	66.7%	27.5%	4.3%	53.1%	39.7%	6.5%
8. Specific drugs are the first option for treatment of psychological and behavioral disorders.	53.6%	39.1%	7.2%	44.2%	37%	17.5%
23. Use nasogastric tube or percutaneous gastrostomy in patients with advanced dementia as a regular feeding route, if dysphagia present.	53.6%	7.7%	7.2%	28.8%	54.8%	16.1%

^a^ Number of each item in the scale. RNs, registered nurses; ANs, assistant nurses; EWs, eldercare workers.

**Table 4:** Association between demographic and educational factors and the knowledge score measured with the UJA Alzheimer’s Care Scale.

Variables	RNs (*n* = 69)		ANs and EWs * (*n* = 292)	
	Score Mean (SD)	*p*-Value ^1^	Score Mean (SD)	*p*-Value
Gender				
Female	19.14 (2.55)	0.779	16.48 (2.95)	0.141
Male	19.50 (1.0)		15.33 (2.19)	
Work experience (years)				
<5	18.00 (2.65)	0.006 ^2^	16.19 (3.09)	0.455
5–14.9	19.46 (2.16)		16.51 (2.79)	
15 years or more	20.33 (2.09)		16.87 (2.93)	
Attendance at courses on dementia (any time)				
Yes	19.41 (2.39)	0.02	16.64 (2.86)	0.01
No	17.25 (2.49)		15.63 (3.01)	
Attendance at courses on dementia (last 3 years)				
Yes	19.84 (1.98)	0.039	17.22 (2.61)	<0.0001
No	18.61 (2.73)		15.96 (2.99)	
Attendance at conferences or meetings (any time)				
Yes	19.59 (2.41)	0.248	17.08 (2.19)	0.01
No	18.88 (2.52)		16.47 (3.12)	

^1^ t-test or ANOVA. ^2^ DMS post hoc test. <5 years vs. 5–14.9 years, *p* = 0.029; <5 years vs. 15 years or more, *p* = 0.002; 5–14.9 years vs. 15 years or more, *p* = 0.227.

**Table 5:** Characteristics of the 24 nursing homes and factors associated with the knowledge score of the staff.

Variables	Nursing Homes Number (%) ^5^	Knowledge Score; Mean (SD)	*p*-Value
Number of residents			
Up to 100	15 (62.5%)	17.16 (2.85)	0.19
More than 100	8 (33.3%)	16.73 (3.22)	
Staff experience ^1^			
1 Very low	4 (16.7%)	15.53 (3.10)	0.001 ^2^
2 Low–	8 (33.3%)	17.42 (2.88)	
3 Medium	9 (37.5%)	16.93 (2.87)	
4 High	3 (12.5%)	17.43 (3.50)	
Percentage of staff that have attended dementia courses in last 3 years ^3^			
1 Low	9 (37.5%)	16.39 (3.11)	0.002 ^4^
2 Medium	13 (54.2%)	17.07 (2.93)	
3 High	2 (8.3%)	18.73 (2.90)	
Ratio of residents per staff member			
Low (Up to 2.90)	10 (41.7%)	16.71 (3.11)	0.19
High (2.91 or more)	9 (37.5%)	17.18 (3.07)	

^1^ Very inexperienced staff: less than 33% of the staff have more than 5 years of experience; inexperienced: >33% of the staff, but less than 66%, have more than 5 years; medium: >66% of the staff have more than 5 years and <33% more than 15 years; Very experienced (>66% of the staff have more than 5 years and >33% more than 15 years). ^2^ Post hoc DMS: 1 vs. 2: *p* < 0.0001; Cohen’s d = 0.65 (CI 95% 0.33–0.97); 1 vs. 3: *p* = 0.003; Cohen’s d = 0.49 (CI 95% 0.17–0.80); 1 vs. 4: *p* = 0.003; Cohen’s d = 0.59 (CI 95% 0.15–1.02) ^3^ Courses attended in last three years: low (<33% of the staff attended courses in the last three years); medium (>33% and >66% of the staff attended courses in the last three years); high (>66% of the staff attended courses in the last three years) ^4^ Post hoc DMS: 1 vs. 2: *p* = 0.046; Cohen’s d = 0.23 (CI 95% 0.0–0.45); 1 vs. 3: *p* = 0.001; Cohen’s d = 0.76 (CI 95 0.29–1.22); 2 vs. 3: *p* = 0.14; Cohen’s d = 0.57 (CI 95% 0.12–1.01). ^5^ Missing data in some variables refer to data not provided by the nursing homes.
